# Enhanced degradation of resin in crude oil-contaminated soil by *Bacillus subtilis* and *Pseudomonas aeruginosa* using surfactants

**DOI:** 10.1098/rsos.250510

**Published:** 2025-08-06

**Authors:** Zhixin Niu, Pengyu Guo, Zhiyun Wang, Ziyao Wang, Taiyu Su

**Affiliations:** ^1^ Key Laboratory of Regional Environment and Eco-remediation, Shenyang University, Shenyang, Liaoning, People’s Republic of China; ^2^ Environmental Department, Shenyang University, Shenyang, People’s Republic of China; ^3^ Longyan University, Longyan, Fujian, People’s Republic of China

**Keywords:** degradation, resins, crude, contaminated, soils

## Abstract

Crude oil inevitably pollutes soil and water during extraction, transport and use. The long-term persistence of resin and asphaltene in the environment poses a threat to ecosystems and human health. The removal of heavy components such as resin is the key to the complete remediation of oil-contaminated soil. Bioremediation, a technology that has been demonstrated to be both environmentally friendly and cost-effective, has attracted significant attention among the oil pollution remediation technologies. In this study, two bacteria, *Bacillus subtilis* and *Pseudomonas aeruginosa*, isolated from soil contaminated with crude oil, were used to investigate the biodegradation of resin in soil. Surfactants, including Tween-80 (TW-80), octadecyltrimethylammonium chloride (ODAC) and sodium dodecylbenzenesulfonate (SDBS), were supplemented to evaluate the effects of surfactant type and concentration on resin degradation. The results showed that total petroleum hydrocarbon content generally decreased in all treatment groups over time, and surfactant addition was effective in supporting bacterial population. In the single surfactant treatments, overall, *Pseudomonas* degraded resin more effectively than *Bacillus*. At the same concentration, SDBS promoted resin degradation more effectively than TW-80 and ODAC at different time points (at the 42nd day: *Bacillus*, 11.8–19.1 g kg^−1^; *Pseudomonas*, 10.7–14.6 g kg^−1^). In the surfactant complex treatment, the combination of ODAC × SDBS resulted in a significant enhancement of the degradation of resin in the soil. However, the presence of TW-80 inhibited the effect of the other surfactants retarding the degradation of resin. This study demonstrated that the combined use of surfactants and degrading bacteria can promote the degradation of heavy fractions in crude oil present in soil. Nevertheless, further exploration is required into the response between surfactants and pollutants and microorganisms as well as the mechanisms of their interaction.

## 1. Introduction

As an important part of global energy, oil plays an indispensable role in industrial production and daily life [1–3]. However, crude oil inevitably pollutes soil and water bodies during its extraction, transportation and use [4,5]. As has been stated [6,7], there is a variety of toxic and hazardous components contained in crude oil. These include polycyclic aromatic hydrocarbons (PAHs), heavy metals and resin and asphaltene heavy fractions. These components are highly environmentally persistent and bioaccumulative. These substances with high molecular weights and complex chemical structures, when introduced into the soil, can lead to the destruction of soil structure, reduce soil permeability and aeration, affect plant growth [8], and negatively affect the structure and function of soil microbial communities, which in turn pose a long-term threat to ecosystems and human health [3,9].

In recent years, a variety of methods for the remediation of oil-contaminated soil have been developed, including physical, chemical and biological approaches [3,10]. Among the numerous oil contamination restoration techniques, bioremediation has attracted considerable attention due to its environmental benefits and cost-effectiveness [11–13]. Cain *et al.* found that a bacterium (CNQ480) screened in the oil spill area of the Gulf of Mexico was able to effectively degrade aromatic hydrocarbons in crude oil [14]. Johnsen showed that *Rhodococcus erythropolis* was able to convert long-chain alkanes into short-chain fatty acids in oil through its metabolic pathway, and this strain was applied in the remediation of oil-contaminated soil in Alaska with good results [15].

The biodegradability of resin or asphaltene in crude oil has been the subject of considerable controversy [16,17]. Uraizee *et al.* concluded that high levels of resin or asphaltene could impede the mass transfer at the contact surface between petroleum hydrocarbons and microorganisms, resulting in extremely difficult degradation such as high molecular weight aromatic hydrocarbons [18]. The study by Chaineau *et al.* also found that the resin and asphaltene were largely unaffected when saturated and aromatic hydrocarbons in crude oil were almost completely degraded by microorganisms [19].

However, many researchers have screened microorganisms that can degrade resin and asphaltene in recent years [3,20]. Hao *et al.* found that heterotrophic, salinophilic bacteria can degrade aromatics and resin [21]. Both Uribe-Alvarez *et al.* and Lavania *et al.* proposed that *Neosartorya fischeri*, which uses resin as its sole carbon source, can remove porphyrins and preferentially degrade aromatic and resin compounds [22,23]. Some bacteria (e.g. *Pseudomonas*, *Achromobacter* and *Rhodococcus*) play a key role in the study of complicated petroleum hydrocarbon degradation, and they can be involved in the biotransformation process of hydrocarbon compounds through their unique physiological adaptations and complex metabolic pathways under different environmental conditions [24,25]. Rahman *et al.* screened five efficient degrading strains of *Micrococcus* sp., *Corynebacterium* sp., *Flavobacterium* sp., *Bacillus* sp. and *Pseudomonas* sp. from 130 petroleum hydrocarbon cultures [26]. Pineda-Flores *et al.* demonstrated the existence of a group of bacteria that degrade heavy oil fractions using resin, etc. as their sole carbon and energy source [16]. Niu *et al.* isolated three bacterial strains from petroleum-contaminated soils of the Liaohe oilfield, which showed good degradation capacity for resin and asphaltene (up to 54.98%) [27]. It has been reported that Proteobacteria was the dominant phylum in all types of petroleum hydrocarbon contaminated sites, with a relative abundance of up to 98.53% in petroleum degrading bacterial lineages [28–31]. As a member of Proteobacteria, *Pseudomonas* is able to degrade quinoline nitrogenous compounds in micelles using oxygenases encoded by the *nidA* gene [32] and can degrade benzothiophene sulfides in micelles through the *dsz* gene cluster (up to 40% removal) [33]. Furthermore, it has been demonstrated that *Bacillus subtilis* can enhance the emulsification efficiency of resin (up to 40%) by secreting surfactin, reduce the critical micelle concentration (CMC), resulting in more than 20% degradation of resin [34,35].

High-viscosity and low-solubility resin in crude oil can lead to poor microbial cell wall permeability, severely limiting microbial action on them [36,37]. However, research in recent years has shown that surfactants can significantly improve the bioavailability of petroleum [38]. As effective surface tension reducing compounds, surfactants can facilitate the mixing of petroleum hydrocarbons with water and increase their solubility in the aqueous phase, making them more accessible and available substrates for microorganisms. Smith and Himmel reported that the addition of the non-ionic surfactant Tween-80 (TW-80) and the anionic surfactant sodium dodecylbenzenesulfonate (SDBS) increased the efficiency of bacterial degradation of crude oil [39]. Cationic surfactants such as dodecylammonium sulfate were found to enhance the degradation of *n*-hexadecane in oil [40]. Furthermore, Chen *et al.* showed that petroleum hydrocarbons can be effectively leached from the soil solid phase to the liquid phase using five different surfactants, of which SDBS performed best in extracting petroleum hydrocarbons from soil with an extraction rate of 81% [41].

At present, there is a paucity of research on the effect of surfactants on the bacterial degradation of resin in crude oil, considering that the removal of resins and asphaltenes is the key to the complete remediation of any type of crude oil-contaminated soil. The two bacteria selected in this study, *Bacillus subtilis* and *Pseudomonas aeruginosa*, were isolated from oil-contaminated soil. They have been demonstrated in many previous studies to have environmental adaptability and targeted degradation ability for resin, and can degrade complex resin into low-toxicity intermediate metabolites, thereby achieving complete mineralization of the resin. Furthermore, the impact of incorporating TW-80, octadecyltrimethylammonium chloride (OADC) and SDBS on the resin degradation process was examined in this experiment. The objective of this article is to evaluate the available potential of different types and concentrations of surfactants to enhance the efficiency of bacterial resin degradation. This will provide a theoretical basis and technical support for the field application of crude oil-contaminated soil bioremediation.

## 2. Results and discussion

### 2.1. The effect of soil texture on microbial degradation of resin

The soil used in this experiment, classified as a silt loam, exhibits a medium texture characterized by relatively low sand content (24%), a predominant silt fraction (56%) and moderate clay content (19%). These textural characteristics critically modulate surfactant-enhanced biodegradation of resin, with divergent mechanisms observed in sandy versus clayey systems. In sand-dominated soils (>70% sand), high hydraulic conductivity enhances surfactant dispersion but accelerates resin migration beyond microbial hotspots, reducing contact efficiency despite favourable oxygen diffusion; column studies have confirmed 23–41% lower degradation of resin fractions compared with finer textures due to contaminant leaching and moisture instability [42]. Conversely, clay-rich matrices (>35% clay) impose dual constraints: chemisorption through cation–π bonding stabilizes resin on surfaces of silt fraction, increasing desorption energy barriers by 18−27 kJ mol^−1^ [43], while their micropore-dominated architecture restricts oxygen diffusion, suppressing monooxygenase activity critical for resin cleavage [44]. Notably, the silt loam utilized herein (24% sand/56% silt/19% clay) demonstrates optimal trade-offs wherein silt-mediated capillary networks facilitate homogeneous surfactant distribution, and its moderate clay content retains contaminants without inducing severe oxygen limitation [45].

### 2.2. Effect of surfactants on bacterial biomass

The biomass of *Bacillus* and *Pseudomona*s decreased gradually with time, with the fastest decline during 0−14 days. In the treatment of surfactants and resins coexisting, the addition of surfactants ODAC and SDBS could slow down the trend of biomass decline. In the single surfactant treatments, the biomass of both bacteria was relatively higher in the SDBS treatment and lower in the TW treatment relative to the control at all time points. Among the surfactant complex treatments, the biomass (42 days) was significantly higher in the ODAC × SDBS treatment than in the other treatments (*Bacillus*, 5.31; *Pseudomonas*, 6.14) (table 1). In resin-free treatments, surfactants (except TW-80) maintained *Bacillus* and *Pseudomonas* cell counts and mitigated the decline in microbial biomass over time compared with controls.

**Table 1.** Effect of surfactants on the biomass of *Bacillus* and *Pseudomonas*. CK, control; TW, Tween-80; ODAC, octadecyltrimethylammonium chloride; SDBS, sodium dodecylbenzenesulfonate. Different letters represent the differences between different treatments at the same concentration and time (*p* < 0.05).

			*Bacillus* (lg(cfu g^−1^))			*Pseudomonas* (lg(cfu g^−1^))		
	**surfacant concentrations**	treatments	14 days	28 days	42 days	14 days	28 days	42 days
	0	CK	4.53 ± 0.366	3.46 ± 0.267	2.75 ± 0.243	4.14 ± 0.148	3.12 ± 0.265	3.04 ± 0.111
treatments with resin added	5 mg kg^−1^	TW	3.63 ± 0.131 c	2.8 ± 0.111 c	2.37 ± 0.099b	3.63 ± 0.103C	2.8 ± 0.157C	2.37 ± 0.183B
		ODAC	4.71 ± 0.139b	3.51 ± 0.769b	2.88 ± 0.195b	4.46 ± 0.283B	3.51 ± 0.167B	2.88 ± 0.226B
		SDBS	5.23 ± 0.141 a	4.89 ± 0.224 a	4.14 ± 0.124a	6.04 ± 0.271A	4.89 ± 0.058A	4.14 ± 0.178A
	10 mg kg^−1−1^	TW	3.91 ± 0.143b	2.98 ± 0.169b	2.37 ± 0.112c	3.74 ± 0.155C	2.76 ± 0.086C	2.37 ± 0.157C
		ODAC	5.57 ± 0.206 a	4.51 ± 0.196 a	3.91 ± 0.162b	5.57 ± 0.273B	4.14 ± 0.158B	3.91 ± 0.288B
		SDBS	5.75 ± 0.195 a	4.9 ± 0.209 a	4.26 ± 0.094 a	6.32 ± 0.164A	5.29 ± 0.258A	4.66 ± 0.158A
	20 mg kg^−1^	TW	3.43 ± 0.099b	2.57 ± 0.149c	2.33 ± 0.088 c	3.43 ± 0.114C	2.55 ± 0.125C	2.26 ± 0.147C
		ODAC	6.11 ± 0.026ab	4.68 ± 0.201b	3.45 ± 0.154b	5.91 ± 0.175AB	4.91 ± 0.162B	3.95 ± 0.252B
		SDBS	6.87 ± 0.121a	5.84 ± 0.119a	4.65 ± 0.209 a	7.23 ± 0.163A	6.43 ± 0.263A	5.62 ± 0.345A
	complex	TW × ODAC	4.35 ± 0.213c	3.42 ± 0.092c	3.08 ± 0.141b	4.13 ± 0.191C	3.42 ± 0.157C	3.17 ± 0.178B
		TW × SDBS	5.87 ± 0.151b	4.58 ± 0.113b	3.26 ± 0.159ab	5.68 ± 0.167B	4.58 ± 0.137B	3.26 ± 0.137B
		ODAC × SDBS	7.51 ± 0.164 a	6.16 ± 0.177a	5.31 ± 0.289a	7.97 ± 0.188A	7.12 ± 0.067A	6.14 ± 0.222A
treatments without resin added	5 mg kg^−1^	TW	3.77 ± 0.213 c	3.09 ± 0.201c	2.57 ± 0.163c	3.82 ± 0.187C	2.95 ± 0.127C	2.55 ± 0.231C
		ODAC	5.05 ± 0.114b	4.14 ± 0.115b	3.27 ± 0.142b	4.58 ± 0.176B	3.71 ± 0.167B	3.26 ± 0.156B
		SDBS	5.54 ± 0.098 a	5.12 ± 0.147a	4.49 ± 0.203a	6.24 ± 0.134A	5.13 ± 0.182A	4.42 ± 0.183A
	10 mg kg^−1^	TW	4.23 ± 0.111 c	3.35 ± 0.159c	2.64 ± 0.182c	4.01 ± 0.118C	2.97 ± 0.154C	2.37 ± 0.155C
		ODAC	5.89 ± 0.223b	4.68 ± 0.168b	4.16 ± 0.179ab	5.89 ± 0.208B	4.45 ± 0.231B	4.16 ± 0.125B
		SDBS	6.18 ± 0.154a	5.29 ± 0.177a	4.63 ± 0.210a	6.61 ± 0.167A	5.55 ± 0.142A	4.87 ± 0.111A
	20 mg kg^−1^	TW	3.58 ± 0.165c	2.46 ± 0.164c	2.13 ± 0.133c	3.65 ± 0.222C	2.76 ± 0.113C	2.46 ± 0.166C
		ODAC	6.39 ± 0.142b	5.12 ± 0.224b	4.14 ± 0.124b	6.16 ± 0.137B	5.13 ± 0.176B	4.45 ± 0.207B
		SDBS	7.23 ± 0.114a	6.22 ± 0.301a	4.98 ± 0.139a	7.45 ± 0.098A	6.84 ± 0.195A	6.01 ± 0.231A
	complex	TW × ODAC	4.58 ± 0.204c	3.81 ± 0.195c	3.35 ± 0.208b	4.52 ± 0.115C	3.71 ± 0.231C	3.47 ± 0.152B
		TW × SDBS	6.08 ± 0.186b	4.89 ± 0.213b	3.93 ± 0.157b	6.18 ± 0.187B	4.84 ± 0.241B	3.63 ± 0.173B
		ODAC × SDBS	7.66 ± 0.113a	6.55 ± 0.173a	5.66 ± 0.182a	8.26 ± 0.169A	7.37 ± 0.182A	6.71 ± 0.189A

In this experiment, microbial growth was more effective in surfactant-only treatments than in surfactant–resin combinations. Without resin, the surfactants ODAC and SDBS exhibited a positive effect on bacterial survival rates. The alkyl chain and benzene ring structure of SDBS, as well as the quaternary ammonium group or alkyl chain of ODAC, could be degraded and utilized as carbon and energy sources by specific types of bacteria. Bacterial enzymes (e.g. oxygenases, desulfonases) progressively decomposed SDBS and ODAC into intermediates (fatty acids, succinic acid) that entered central metabolic pathways, promoting growth [46]. When resin and surfactants coexisted, surfactants could solubilize resin components through micelle formation, releasing bioavailable monomers. For instance, surfactin solubilized resin to release styrene, a carbon source for *Bacillus subtilis* that increased microbial community richness by 40% [47]. Similarly, Wei *et al.* used high-performance liquid chromatography to quantify a 3.2-fold increase in terephthalic acid release from polyethylene terephthalate resin by sophorolipids; this became *Ideonella sakaiensis*’s primary carbon source, elevating cell density by 50% [48]. Additionally, surfactants like SDBS and TW-80 bolstered microbial antioxidant defenscs. In *Pseudomonas* exposed to epoxy resin, the intracellular content of malondialdehyde (a lipid peroxidation marker) decreased by 28%, while superoxide dismutase activity rose 1.7-fold, increasing cell viability from 68 to 89% [49].

**Figure 1.**
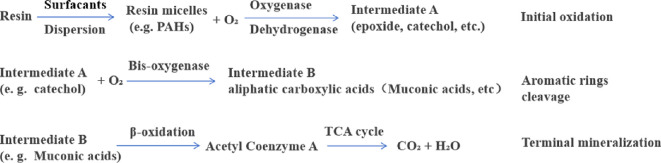
Microbial degradation reactions for resin.

Resin and the intermediate metabolites produced in this experiment showed high toxic effects on microorganisms. The basic reactions of resin degradation by microorganisms are shown in figure 1. It has been observed that resins or asphaltenes are capable of forming macromolecular aggregates with high molecular mass, high viscosity and low solubility through hydrogen bonding and van der Waals forces [4,50–53]. This structure can lead to the adsorption and deposition of petroleum hydrocarbons on the surface of microbial cells, which can affect the attachment of microorganisms and the formation of biofilms, interfere with the normal function of cells and even cause cell death [54,55]. In addition, it has been hypothesized that resin and its metabolic intermediates may interact with functional groups of microbial cell walls, thereby altering cell wall permeability and affecting microbial metabolic activities [9,56]. Despite the fact that the chemical structure and toxicity of resin in crude oil are not conducive to the growth of organisms [33,52,57], the results of the present experiments demonstrated the degradation of resin using two strains of bacteria screened from crude oil-contaminated soil. Microorganisms can reduce the viscosity of thick oil and improve the fluidity of crude oil through secreted enzymes or the production of biosurfactants, increasing the bioavailability of hydrophobic components [49,58,59]. The degradation pathways of these microorganisms generally include the following: (i) ring-opening degradation of PAHs; (ii) degradation of long-chain *n*-alkanes; and (iii) ring opening of heterocyclic compounds to remove heteroatoms [17,57,60,61].

Furthermore, the microbial degradation of resin in this experiment was difficult (below 70%), which is consistent with the findings of Roy *et al.* and Li *et al.*, who concluded that although studies have been conducted to screen some microbial strains that can degrade resin or asphaltene, including bacteria, actinomycetes and fungi, the degradation rate of the currently strains is generally not high. Further studies are needed to explore the mechanisms of the effects of resin on microbial biometabolism and populations [62,63].

### 2.3. Effect of surfactants on the contents of total petroleum hydrocarbons, resin, aromatic hydrocarbons and saturated hydrocarbons

#### 2.3.1. Changes in total petroleum hydrocarbon contents

**Figure 2.**
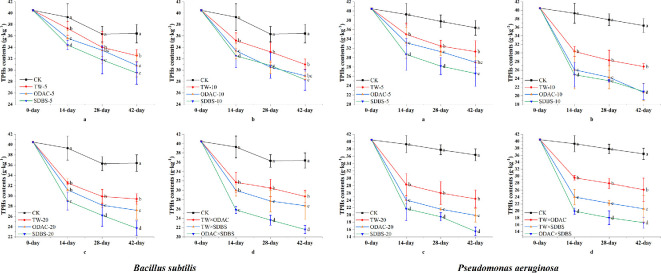
Changes in total petroleum hydrocarbon contents of soil in single and complex surfactant treatment groups of *Bacillus* and *Pseudomonas*. CK, control; TW, Tween-80; ODAC, octadecyltrimethylammonium chloride; SDBS, sodium dodecylbenzenesulfonate. The lowercase letters on the error bars indicate that the values in different surfactant treatments at the same time are significantly different at *p* < 0.05.

The total petroleum hydrocarbon (TPH) contents of all treatment groups generally decreased with time; between 14 and 42 days, the TPH contents of SDBS-treated groups were significantly lower than those of TW and ODAC in the different concentrations of surfactant treatments of the two bacteria, which were 23.7 g kg^−1^ (*Bacillus*, 42 days), 15.5 g kg ^−1^ (*Pseudomonas*, 42 days). Among the complex treatments, the ODAC × SDBS treatment had the lowest TPH contents (*Bacillus*, 21.6–25.8 g kg^−1^; *Pseudomonas*, 19.9–16.2 g kg^−1^). In addition, the combination of TW and ODAC did not result in a significant decrease in TPH contents compared with the two surfactants alone (figure 2).

#### 2.3.2. Changes in resin contents

**Figure 3.**
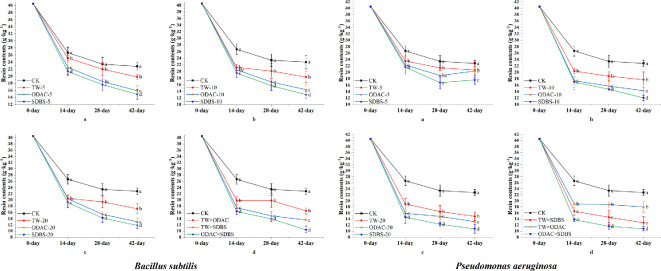
Changes in resin contents of soil in single and complex surfactant treatment groups of *Bacillus* and *Pseudomonas*. CK, control; TW, Tween-80; ODAC, octadecyltrimethylammonium chloride; SDBS, sodium dodecylbenzenesulfonate. The lowercase letters on the error bars indicate that the values in different surfactant treatments at the same time are significantly different at *p* < 0.05.

Both surfactant additions promoted bacterial degradation of the resin, and the resin contents in the soil basically decreased with increasing time and surfactant concentration. At the same concentration, SDBS promoted resin degradation more effectively than TW and ODAC at different time points (SDBS-20: *Bacillus*, 11.8–19.1 g kg^−1^; *Pseudomonas*, 10.7–14.6 g kg^−1^) (*p* < 0.05). In general, *Pseudomonas* exhibited superior resin-degradation capability compared with *Bacillus* for a given type and concentration of surfactant (figure 3).

#### 2.3.3. Changes in aromatic hydrocarbon contents

**Figure 4.**
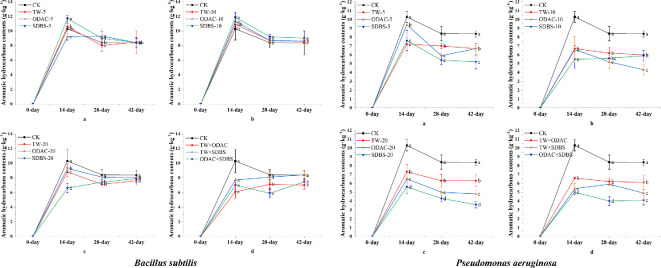
Changes in aromatic hydrocarbon contents of soil in single and complex surfactant treatment groups of *Bacillus* and *Pseudomonas*. CK, control; TW, Tween-80; ODAC, octadecyltrimethylammonium chloride; SDBS, sodium dodecylbenzenesulfonate. The lowercase letters on the error bars indicate that the values in different surfactant treatments at the same time are significantly different at *p* < 0.05.

Aromatic hydrocarbons in the *Pseudomonas* treatments showed lower levels than the *Bacillus* treatments at all time points and at different concentrations. The effect of different types of surfactants on the aromatic hydrocarbon content of *Bacillus* treatments was less pronounced than that of *Pseudomonas* treatments. The highest aromatic hydrocarbons in the *Bacillus* treatments were found in the 10 mg l^−1^ surfactant treatments (10.9−11.9 g kg^−1^) at the 14th day, whereas the highest aromatic hydrocarbons in the *Pseudomonas* treatments appeared in TW-5 (7.2 g kg^−1^). The addition of the complex surfactants caused an irregular variation in the aromatic hydrocarbon contents in the *Bacillus* treatments over time. The aromatic hydrocarbon content in the *Pseudomonas* treatments was ODAC × SDBS, TW × SDBS, and TW × ODAC in descending order of magnitude (figure 4).

#### 2.3.4. Changes in saturated hydrocarbon content

**Figure 5.**
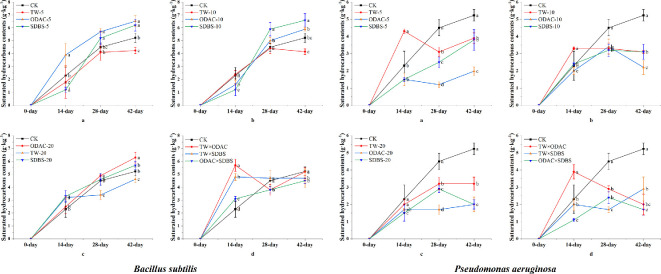
Changes in saturated hydrocarbon contents of soil in single and complex surfactant treatment groups of *Bacillus* and *Pseudomonas*. CK control; TW, Tween-80; ODAC, octadecyltrimethylammonium chloride; SDBS, sodium dodecylbenzenesulfonate. The lowercase letters on the error bars indicate that the values in different surfactant treatments at the same time are significantly different at *p* < 0.05.

Saturated hydrocarbon contents generally increased in *Bacillus* with the addition of single surfactant treatments (except TW-10) (figure 5). At the 42nd day, the highest saturated hydrocarbon content was found in the SDBS-5 and ODAC-10 treatments (6.52 and 6.59 g kg^−1^, respectively). In the *Pseudomonas* treatments, saturated hydrocarbons displayed irregular variations. The lowest values were observed in the SDBS-5 treatment (1.2−1.98 g kg^−1^), in comparison with the other treatments. In the complex treatment groups, the highest saturated hydrocarbon content was found in the TW × SDBS at the 14th day (*Bacillus*, 5.8 g kg^−1^; *Pseudomonas*, 3.7 g kg^−1^). In contrast, the ODAC × SDBS treatment exhibited the lowest saturated hydrocarbon content at 42nd day (*Bacillus*, 4.5 g kg^−1^; *Pseudomonas*, 1.6 g kg^−1^). The contents of saturated hydrocarbons in *Bacillus* treatments were generally higher than those in *Pseudomonas* treatments, particularly in TW × SDBS and ODAC × SDBS treatments.

#### 2.3.5. Correlation analysis of factors

**Figure 6.**
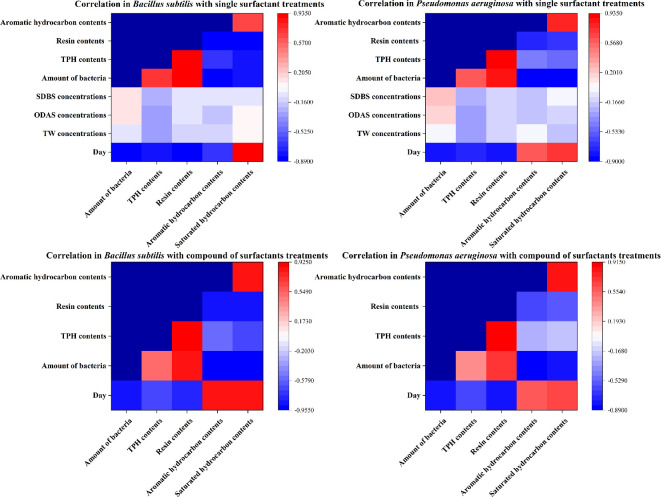
Correlation analysis of microbial biomass, total petroleum hydrocarbons, resin, aromatic hydrocarbons and saturated hydrocarbons contents in single and complex surfactants treatments.

In *Bacillus* treatments, bacterial biomass, TPHs and resin were negatively correlated with time. Conversely, saturated hydrocarbon contents increased with time. Bacterial biomass was negatively correlated with the content of the different metabolites in the *Pseudomonas* treatments, which was particularly pronounced in the composite treatment. Surfactant TW inhibited the biomass of microorganisms and hindered the degradation of resin in the complex treatments, whereas the use of surfactant SDBS, either alone or in combination, resulted in the promotion of microbial growth and a significant increase in the degradation rate of resin and aromatic hydrocarbons (figure 6).

Surfactants have exhibited considerable potential in enhancing the efficiency of microbial utilization of organic pollutants [64–66]. Surfactants possess a variety of properties, including emulsifying, wetting, dispersing, solubilizing and decontaminating. They contain polar and non-polar groups, which have the capacity to reduce the surface tension between different interfaces of liquids, solids and gases [67,68].

The structure of surfactants can elute petroleum hydrocarbons adsorbed on soil particles, thereby forming small oil droplets of micelles to be dispersed stably in the soil aqueous phase, and increase the mobility of petroleum hydrocarbons as well as the microbial contact area, thus increasing the degradation rate of petroleum hydrocarbons [69–71]. Asadollahi *et al.* showed that the addition of lipopeptide surfactant reduced the surface tension of the resin to 30.2 mN m^−1^ after 48 h under optimal conditions, with a CMC of 23.4 mg l^−1^, which significantly increased the ability of *Bacillus cereus* to degrade the resin (up to 40%) [72]. Surfactants can improve microbial cell surface hydrophobicity (CSH) and cell membrane permeability [73,74]. Studies have shown that lipopeptide surfactants can improve CSH during the degradation of petroleum hydrocarbons by *Geobacillus stearothermophilus* A-2, and the increase in CSH makes it easier for contaminants to attach on the bacterial surface, thus regulating cell, hydrophobic matrix and the interaction between cells, hydrophobic matrix and contaminants, which facilitates the transfer of contaminants from micelles to the interior of microbial cells [75]. Moreover, microbial cell membranes, composed of a phospholipid bilayer, exhibit structural similarity to surfactants. Consequently, surfactants have the capacity to interact with microbial cell membranes and modify their structure and function, including increasing extracellular polymer (EPS) secretion, biofilm roughness and biofilm adhesion and affecting the mass transfer and permeability of the cell membrane [51,76].

In this experiment, TW-80 showed inhibition of bacterial biomass. This is in line with the study of Niu *et al.*, who found that TW-80 significantly inhibited the growth of microorganisms (A2016) in a study of degradation of resin and asphaltene under laboratory incubation conditions [27]. The study by Nielsen *et al.* also revealed that TW-80 can influence microbial growth and biofilm formation, thereby impeding the proliferation of *Pseudomonas fluorescens* and reducing the capacity of *Listeria monocytogenes* to form biofilms [77]. Also, TW-80 has been shown to induce permeability alterations in the membrane system (vesicles composed of cardiolipin and phosphatidylglycerol) of specific microorganisms, which in turn affects microbial growth and metabolic processes [78,79].

In the present study, the enhancement of bacterial degradation of resin by different concentrations of the three surfactants alone and in combination was found to be significantly different. In comparison with the other two surfactants, the use of SDBS and ODAC markedly promotes microbial growth and increases the degradation rate of petroleum hydrocarbons. This is basically in accordance with the study of Chen *et al.* who compared the effects of five types of surfactants, namely, TW-80, cocamidopropyl betaine (CAB), rhamnogalactan (RHA), SDBS and ODAC, on the microbial electrochemical remediation of oil-contaminated soil, and found that the soil petroleum hydrocarbon extraction effect in the order of SDBS (81%) > CAB (76%) > ODAC (74%) > TW-80 (71%) > RHA (50%) [41]. As Zhang *et al.* and Pan *et al.* have established, the treatment of microbial cells with SDBS or ODAC resulted in a significant increase in the content of unsaturated fatty acids, which strengthened the fluidity of the cell membranes and facilitated the rate of contaminant transmembrane transport [30,80].

Furthermore, the degradation of resin and aromatic hydrocarbons was considerably enhanced in the SDBS × ODAC treatments in this experiment. It has been proved that a mixture of SDBS and ODAC in suitable proportions can significantly improve micelle formation, surface activity, dynamic adsorption, foaming, emulsification and cell permeability of a single surfactant [81,82]. The utilization of SDBS in combination with non-ionic surfactants (e.g. tralatone-100, TW-80) has been found to result in a significant escalation in the leaching rate of persistent organic pollutants that are firmly bound to soil particles [31].

It is evident that the application of surfactants to enhance the degradation of resin in soil contaminated with crude oil needs to take into account the variations in surfactants and the physiological characteristics of microbial populations. A more profound investigation is required to ascertain the intricate relationship between surfactants and microorganisms, as well as the matching and response between these two factors.

#### 2.3.6. Description of experimental design and results

**Figure 7.**
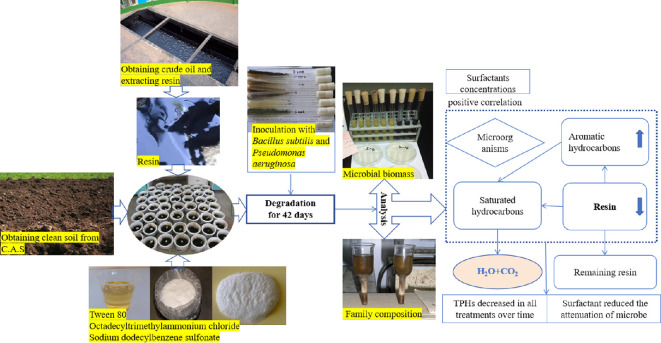
Schematic diagram of experimental design and main results.

The design and procedure of this experiment as well as the primary results are illustrated concisely in figure 7.

## 3. Conclusions

This study investigated the effect of different concentrations of surfactants (TW-80, OADC and SDBS) on the degradation of resin by bacteria (*Bacillus* and *Pseudomonas*). In summary, *Pseudomonas* exhibited superior degradation of resin compared with *Bacillus*, under equivalent conditions with regard to type and concentration of surfactant. In the single surfactant treatments, SDBS enhanced bacterial growth and resin degradation more effectively than the other two surfactants. The presence of TW-80 in the complex treatments limited the microbial growth and hindered the resin degradation to varying degrees, whereas the degradation of petroleum hydrocarbons in the soil was well enhanced in the ODAC × SDBS treatments. It is acknowledged that the composition of resins in diverse crude oils and the heterogeneity of soil types may introduce discrepancies in the degree of contamination and characteristics of resin-contaminated soils simulated in this experiment, relative to actual crude oil-contaminated site soils. Consequently, artificially spiked soils may yield different remediation or detection outcomes compared with real-world contaminated soils. Nonetheless, it is posited that the elimination of resins and asphaltene constitutes a pivotal phase for achieving complete remediation of crude oil-contaminated soils, irrespective of the specific type of crude oil involved. The findings of this study suggest that the combined utilization of surfactants and bacteria represents a highly effective strategy for enhancing the biodegradation efficiency of heavy fractions in soils contaminated with crude oil. However, further exploration is required to understand the interaction mechanisms among surfactants, pollutants and microorganisms, as well as the matching and response relationships between surfactant properties and microbial characteristics in practical applications.

## 4. Experimental

### 4.1. Material and methods

#### 4.1.1. Test soils and extraction of resin

The soil used in this experiment was obtained from the Shenyang Ecological Station of the Chinese Academy of Sciences, CAS. Total organic matter content, total phosphorus, total nitrogen, cation exchange, pH and soil texture were determined according to the International Organization for Standardization. Soil samples were air-dried at 40°C, homogenized and sieved (≤2 mm) before analysis; for organic matter, inorganic carbon was removed by 1 M HCl pretreatment followed by combustion at 900°C with evolved CO_₂_ quantified via infrared detection [83]. Total nitrogen and phosphorus were determined after Kjeldahl digestion and sulfuric acid–hydrogen peroxide digestion respectively, using automated flow-injection analysis [84,85]. Cation exchange capacity was measured by saturating soil with 0.1 M BaCl₂, washing with ethanol and quantifying displaced Ba²^+^ through inductively coupled plasma optical emission spectrometry [86]. pH was potentiometrically measured in 1:5 (w/v) soil–water suspensions after 2 h equilibration [87]. Mechanical composition required sequential pretreatment with 30% H_₂_O_₂_ (organic matter removal) and 10% HCl (carbonate dissolution), followed by sodium hexametaphosphate dispersion, wet-sieving for sand fractions (63 μm–2mm), and sedimentation for silt/clay fractions at 25°C using Stoke’s law and analytical replicates included throughout for quality assurance [88]. The physical and chemical properties are shown in table 2. Crude oil samples were gathered from the Walian Oil Extraction Plant, Liaohe Oilfield, Liaoning Province, China. The analysis of the group components of crude oil was conducted by extraction method [89,90]. Crude oil samples were mixed with *n*-heptane at 1:40 (v/v) and dispersed by ultrasonication for 10 min and then left to stand for 24 h. The asphaltenes were separated by filtration, then excess anhydrous ethanol was added to the filtrate, the precipitate was collected by centrifugation (8000 r.p.m., 20 min), and the precipitate was dried under vacuum (40°C, 24 h) to obtain the resin. Molecular weight distribution of resin was analysed by gel permeation chromatography (tetrahydrofuran eluent at 40°C and polystyrene calibration) [91]. Density was measured through oscillating U-tube densitometry (at 20°C); boiling point distribution was determined by high-temperature simulated distillation (GC-FID); surface tension was quantified using pendant drop tensiometry (at 25°C) [92]. Elemental composition (C, H, N, S, O) was obtained through combustion analysis and X-ray photoelectron spectroscopy for surface stoichiometry [93]. Functional groups were identified by Fourier-transform infrared spectroscopy (attenuated total reflectance; 400−4000 cm^−1^) and ^1^H/^13^C NMR (600 MHz, CDCl_3_ solvent) with all samples degassed at 60°C under N_2_ for 24 h to remove volatiles before analysis [94]. The physical and chemical properties of resin samples were obtained by ASTM standard methods (table 3). Although the resin used in the experiments underwent an extraction process, its state and structure may differ from that of resin naturally present in crude oil. This could potentially lead to differences in the microbial degradation process and outcomes compared with the degradation of resin in actual crude oil-contaminated soil environments. From the perspective of content change analysis, this study essentially neglects the influence and limitations of the extraction process.

**Table 2.** Physical and chemical properties of test soils.

	total organic carbon (g kg^−1^)	total phosphorus (g kg^−1^)	total nitrogen (g kg^−1^)	cation exchange capacity (mmol kg^−1^)	pH	particle size distribution (%)		
						<2 μm	2−50 μm	50−2000 μm
experimental soil	36.69	0.81	1.46	8.77	8.5	19.8	56.1	24.1

**Table 3.** Physical and chemical properties of resin samples.

	molecular weight (g mol^−1^)	density (g cm^−3^)	boiling point (°C)	interfacial tension (oil/water) (mN m^−1^)	ratios of major elements	functional groups
resin samples	500−3000	1.0−1.2	350−600	33.21−35.68	C (80–85%), H (8–10%), S (0.5–5%), N (0.5–2%), O (1–5%) and trace metals	methylene, aromatic rings, carboxylic groups, thiocycle, etc.

#### 4.1.2. Microbiological preparation

Two bacterial strains (*Bacillus* and *Pseudomonas*) screened from oil-contaminated soil in Liaohe Oilfield were obtained from the bacterial library of Shenyang Institute of Applied Ecology, CAS. Bacteria were identified using 16S rRNA gene sequencing. The 16S rRNA gene fragment of bacteria was amplified using universal primers (27F/1492R). The sequences were compared for similarity to identify the bacteria through the NCBI database (https://www.ncbi.nlm.nih.gov). The strains were subsequently inoculated into beef paste peptone medium (Huankai Microbial, China) for shaker culture (150 r.p.m., 30°C). After 3 days, the enriched bacteria were collected by centrifugation (8000 r.p.m., 2 min) and dissolved in sterile water to prepare bacterial suspensions.

#### 4.1.3. Experimental design

The clean soil was sieved (0.25 mm pore size) and packed into pots of 500 g of soil each. The resin was dissolved with trichloromethane and sprayed evenly into the soil with constant stirring and equilibrated for 30 days to achieve a resin content of 48.0 g kg^−1^ in the soil.

Surfactants (chemical pure, Ruibio, China) were selected as TW-80, SDBS and ODAC. Before the inoculation with bacteria, the surfactants were added to the soil with thorough mixing and blending. The single surfactant treatments were added at concentrations of 5, 10 and 20 mg kg^−1^ (labelled as TW, SDBS and ODAC concentration values), respectively, and the complex surfactant treatments were TW × SDBS, TW × ODAC, and SDBS × ODAC (each surfactant concentration was 20 mg kg^−1^), and the total surfactant load was double what was present in the highest single surfactant dose (40 mg kg^−1^). Different treatments were equilibrated for 7 days. The recovery of resin in treatments with different concentrations of surfactants is shown in table 4. Bacterial suspensions were introduced into each treatment at the dosage of *Bacillus*, 6.31 × 10^11^ cfu g^−1^; *Pseudomonas*, 5.01 × 10^10^ cfu g^−1^. The temperature was maintained 25–30°C and the soil moisture content was 20–30%. The incubation was carried out for 42 days, with three replicates of each treatment. Samples were taken at 14, 28 and 42 days for analysis of microbial biomass and petroleum hydrocarbon compositions and group compositions.

**Table 4.** Recovery rates of resin in treatments with different concentrations of surfactants.

surfacant concentrations (mg kg^−1^)	surfacant treatments	recovery rates (%)
0	CK	84.38 ± 0.981d
5	TW	86.46 ± 0.858abcd
	ODAC	85.63 ± 0.941cd
	SDBS	86.67 ± 0.908abcd
10	TW	87.71 ± 1.284abc
	ODAC	87.08 ± 1.436abcd
	SDBS	87.71 ± 0.981abc
20	TW	89.17 ± 0.982 a
	ODAC	88.75 ± 2.198ab
	SDBS	88.96 ± 2.429ab
	TW × ODAC	88.33 ± 0.695abc
	TW × SDBS	87.08 ± 1.743abcd
	ODAC × SDBS	86.25 ± 1.901bcd

### 4.2. Experimental methods

#### 4.2.1. Microbial biomass analysis

Microbial biomass in soil was determined by plate counting method. The soil sample solutions were diluted into different gradients of bacterial suspensions according to 10 times. The plates were then coated with 4−7 times diluted bacterial suspensions, with three parallel samples being prepared for each dilution concentration. The inoculated medium was placed in a constant temperature incubator and incubated at 35 ℃ for 3 days and then counted.

#### 4.2.2. Group composition analysis

The air-dried soil samples were ground and passed through a 0.25 mm pore size sieve, 5.0 g of sieved soil samples were taken and mixed with 100 ml of chloroform, this solution was filtered through a funnel stuffed with skimmed cotton wool into a weighing flask [89]. The filtrate was evaporated solvent to dryness (temperature ≤ 40°C), about 30 ml of *n*-hexane was added and dissolved by sonication. The solution was concentrated to 2−3 ml using a rotary evaporator, and then passed over a chromatography column (3–4 g chromatographic silica gel, 2−3 g neutral alumina). The saturated hydrocarbons were separated by washing with *n*-hexane for six times, with each wash comprising 3−5 ml of the solvent. The aromatic hydrocarbons were separated by washing with a solvent mixture of dichloromethane and *n*-hexane (2:1, v/v) for four times at 3−5 ml each time. Finally, the resin was washed with 10 ml of anhydrous ethanol and 10 ml of chloroform. The saturated hydrocarbons, aromatic hydrocarbons and resin obtained from the above separation were volatilized to dry weighing (≤40°C). The extraction recovery of the group components was 85%.

### 4.3. Statistical analysis

All data obtained in this study were analysed by one-way ANOVA using SPSS statistical software (SPSS Inc., Chicago) with a significant level of difference of 0.05. All graphs were plotted using Origin (Pro) software (2022, OriginLab).

## Data Availability

The original contributions presented in this study are included in the article. Further inquiries can be directed to the corresponding author.
